# Efficacy of Cerebrolysin Treatment as an Add-On Therapy to Mechanical Thrombectomy in Patients With Acute Ischemic Stroke Due to Large Vessel Occlusion: Study Protocol for a Prospective, Open Label, Single-Center Study With 12 Months of Follow-Up

**DOI:** 10.3389/fneur.2022.910697

**Published:** 2022-07-04

**Authors:** Jacek Staszewski, Adam Stȩpień, Renata Piusińska-Macoch, Aleksander Dȩbiec, Katarzyna Gniadek-Olejniczak, Emilia Frankowska, Artur Maliborski, Zoltan Chadaide, David Balo, Beata Król, Rafael Namias, George Harston, Józef Mróz, Piotr Piasecki

**Affiliations:** ^1^Clinic of Neurology, Military Institute of Medicine, Warsaw, Poland; ^2^Neurorehabilitation Clinic, Military Institute of Medicine, Warsaw, Poland; ^3^Department of Radiology, Military Institute of Medicine, Warsaw, Poland; ^4^Brainomix Ltd., and Oxford University Hospitals NHSFT, Oxford, United Kingdom

**Keywords:** Cerebrolysin, add-on therapy, mechanical thrombectomy, ischemic stroke, cytoprotection

## Abstract

This study is designed to determine the efficacy of Cerebrolysin treatment as an add-on therapy to mechanical thrombectomy (MT) in reducing global disability in subjects with acute ischemic stroke (AIS). We have planned a single center, prospective, open-label, single-arm study with a 12-month follow-up of 50 patients with moderate to severe AIS, with a small established infarct core and with good collateral circulation who achieve significant reperfusion following MT and who receive additional Cerebrolysin within 8 h of stroke onset compared to 50 historical controls treated with MT alone, matched for age, clinical severity, occlusion location, baseline perfusion lesion volume, onset to reperfusion time, and use of iv thrombolytic therapy. The primary outcome measure will be the overall proportion of subjects receiving Cerebrolysin compared to the control group experiencing a favorable functional outcome (by modified Rankin Scale 0–2) at 90 days, following stroke onset. The secondary objectives are to determine the efficacy of Cerebrolysin as compared to the control group in reducing the risk of symptomatic secondary hemorrhagic transformation, improving neurological outcomes (NIHSS 0–2 at day 7, day 30, and 90), reducing mortality rates (over the 90-day and 12 months study period), and improving: activities of daily living (by Barthel Index), health-related quality of life (EQ-5D-5L) assessed at day 30, 90, and at 12 months. The other measures of efficacy in the Cerebrolysin group will include: assessment of final stroke volume and penumbral salvage (measured by CT/CTP at 30 days) and its change compared to baseline volume, changes over time in language function (by the 15-item Boston Naming Test), hemispatial neglect (by line bisection test), global cognitive function (by The Montreal Cognitive Assessment), and depression (by Hamilton Depression Rating Scale) between day 30 and day 90 assessments). The patients will receive 30 ml of Cerebrolysin within 8 h of AIS stroke onset and continue treatment once daily until day 21 (first cycle) and they will receive a second cycle of treatment (30 ml/d for 21 days given in the Outpatient Department or Neurorehabilitation Clinic) from day 69 to 90.

## Introduction

The recent endovascular stroke trials have established a new paradigm for acute ischemic stroke (AIS) treatment showing that mechanical thrombectomy (MT) within 6 h of stroke due to large vessel occlusion (LVO) significantly reduces the mortality rate and improves clinical outcomes (1). These positive results were not only driven by technical advances and improved endovascular devices, but also by a refinement of patient selection criteria, including the use of perfusion and collaterals status (2). Perfusion imaging can be used in late-window patients with LVO presenting 6–24 h after symptom onset or with unknown stroke onset in select patients with a small ischemic core and a large penumbral zone, which is a predictor of good neurologic outcome after recanalization (3). The assessment of collateral flow may also be necessary in the extended window to distinguish ischemia with fast or slow progression to irreversibility. Advanced imaging is currently not recommended to select patients presenting from 0 to 6 h from the time last known well; however it may help predict the early outcome of MT in individual patients, e.g., higher collateral grades have been associated with better recanalization rates, while patients with poor collaterals tend to have worse outcome even with complete recanalization of parent occlusion (4).

Despite these advances, the rates of functional independence at 3 months following MT performed in both the early and late time window, in clinical trials, or in clinical practice are far from satisfactory (14–58%) compared with the high rates of recanalization (60–90%) (5, 6). Efficacy of MT is related to multiple reasons e.g., time from stroke onset to reperfusion (OTR), secondary intracerebral hemorrhages (ICH) due to reperfusion injury, and the lack of successful recanalization or reflow. The mechanism underlying *no-reflow phenomenon* has not been fully established, but there is now class II evidence of no-reflow in human stroke (7). However, this phenomenon is probably independent of MT technique, can persist despite proximal recanalization, and results from altered microvascular circulation, proinflammatory state, and thrombosis, and represents a potential therapeutic target (8). Another important aspect that may result in increased morbidity is related to periprocedural complications of MT and include arterial wall damage and dissections, ICH, distal embolism, and vasospasm. Beside of early outcomes, MT also results in favorable outcomes at long-term follow-up compared to standard medical treatment alone. However, prospective follow-up data from MT trials indicate that 3-month MT benefits will likely translate into lower long-term mortality and disability (with about 31–36% of functionally disabled patients 5 years post-stroke), but these group-level data by no means guarantee maintenance of 3-month positive outcome for individual patients (9). Several critical factors operate in the post-stroke period that can influence long-term recovery of the patient, and the benefits depend on continuing and optimizing stroke prevention, access to rehabilitation, and post-stroke depression care.

The concepts of cytoprotection and neurorecovery have been researched in many preclinical and clinical settings in acute or chronic stroke in the past decades. Despite promising experience with different agents, suggesting that they can impede the evolution of penumbra into core, reduce reperfusion injury, improve tissue reperfusion, brain plasticity, or neurogenesis, these strategies have not been recommended in humans so far (10). Number of reasons could explain this outcome e.g., lack of persistent recanalization or reperfusion, too late intervention, suboptimal dosage, single mechanisms of action targeting only one mechanism in ischemic cascade, or one type of cells in neurovascular unit (NVU), mismatch between animals and humans, and/or unrobust methodological approaches that resulted in inconsistent evidence (11, 12). Therefore, the focus of modern stroke treatment should be shifted from a neuroprotection to neurovascular protection approach because elements of NVU show differential vulnerability evolving over differing time scales and their roles are crucial in blood-brain-barrier (BBB) regulation, cell preservation, inflammatory immune response during or after AIS (13, 14).

It can be anticipated that cytoprotection in patients who achieve early successful recanalization may promote reperfusion, protect or reduce the consequences of reperfusion damage, and improve early outcome (15, 16). Additionally, effective management should target enhancing collateral circulation and preventing the no-reflow phenomena and hemorrhagic transformation after AIS. Recent results of ESCAPE-NA1 study on nerinetide, a novel neuroprotection agent which promotes cell survival and disrupts toxic cell signals following ischemia, revealed that early treatment in patients without large infarct core and with effective collateral circulation undergoing reperfusion therapy was feasible (17). Although the results showed no significant differences between the nerinetide and the placebo groups, a significant positive effect of nerinetide (improved functional outcome, reduced mortality, decreased infarction volumes) was observed in patients not receiving bridging recombinant tissue plasminogen activator (r-tPA). These findings suggested a probable drug–drug interaction and nerinetide as an add-on therapy to MT is currently being investigated in another randomized clinical trial (RCT) (18).

Motor rehabilitation in acute and post-acute stroke is a standard of care because it allows for functional improvements; however, it may lack the potential of full recovery for patients with large infarcts and there remain significant concerns for long-standing disability and neurological deficits in these patients (19). Although stroke neurorestorative strategies combined with rehabilitation may improve the long-term neurological outcome and quality of life, however clarification of their roles in mediating post-stroke neurorecovery warrants further investigations (20, 21). Preclinical stroke models demonstrate positive responses to different cytoprotective agents with improved cognitive and motor function, therefore, as stated in the Action Plan for Stroke in Europe 2018–2030, continuing to bridge the translational gap between basic and clinical stroke research is vital for the development of effective treatment (22, 23).

Cerebrolysin is a neurotrophic peptidergic preparation with broad pharmacological properties (24). It displays multifactorial cytoprotective properties, improves cellular survival, inhibits glutamate excitotoxicity, free radical formation, and proinflammatory mediators (e.g., TNF-α, IL-1β, IL-6, and NF-κB) (25). It mimics the action of endogenous neurotrophic factors in brain protection and recovery (26). Cerebrolysin has been the subject of many animal and *in vitro* studies, the majority of which have yielded encouraging results in terms of pleiotropic and multimodal activity (27). However, only a few preclinical and clinical studies tested the efficacy of Cerebrolysin as an add-on therapy to MT and/or r-tPA in AIS so far. Recent *in vitro* study showed that Cerebrolysin protects BBB and has a therapeutic effect on r-tPA and fibrin-impaired cerebral endothelial cell permeability by reducing proinflammatory and procoagulation proteins and by elevating tight junction proteins, therefore reducing hemorrhagic transformation, a major safety concern especially for patients at the end of r-tPA time-window (28). In the rat transient middle cerebral artery occlusion model, administration of Cerebrolysin at 3 h post-ischemia reduced infarct volume and promoted long-term functional recovery by reducing neuroinflammation *via* the activation of CREB/PGC-1α pathway and by inhibiting free radical formation (29, 30). In the CERE-LYSE study, the combination of Cerebrolysin (30 mL/day, for 10 days) with r-tPA in humans was safe although it did not significantly improve functional outcome in the modified Rankin Scale (mRS) at 3 months, but in the National Institutes of Health Stroke Scale (NIHSS) responder analysis (secondary outcome measure) significantly more patients had an improvement of 6 or more points after two-, five-, 10, and 30 days in the Cerebrolysin group (31). This advantage was most pronounced in the most severely affected patients (NIHSS 15-25). In a recently published pilot trial, 44 severe stroke patients (NIHSS>8) were randomized to receive Cerebrolysin (30 ml/day, for 14–21 days, *n* = 23) or standard therapy (*n* = 21) following futile reperfusion therapy (r-tPA and/or MT). There was no statistically significant difference in the distribution of clinical outcomes between groups at 90 days. There was, however, a trend for a more favorable outcome (mRS 0-3) at month 12 in the Cerebrolysin group compared with controls (respectively, 70 vs. 48% of the subjects; *p* = 0.1) and a statistically significant reduction of hemorrhagic transformation rates in patients receiving Cerebrolysin (13 vs. 38%, *p* < 0.05) (32). No safety issues were found. In a large (*n* = 1070) randomized, placebo-controlled study in AIS patients not receiving reperfusion therapy, there was no significant difference in confirmatory end-point (combined global directional test of mRS, Barthel Index [BI], NIHSS at 90 days) between the Cerebrolysin (30 ml/daily for 10 days, started within 12 h after stroke onset) and control groups; however the subjects did not receive a similar level of rehabilitation care (33). A *post hoc* analysis showed a trend in favor of Cerebrolysin in patients with severe stroke (NIHSS >12) with a significant difference in favorable NIHSS change from baseline (OR 1.27; CI lower bound, 0.97, *p* = 0.04). In this subgroup, the cumulative mortality by 90 days was 20.2% in the placebo and 10.5% in the Cerebrolysin group (HR 1.9661; CI lower bound, 1.0013, *p* = 0.02). The results of several other studies also showed positive treatment effects in the severely affected ischemic stroke population and in some patients with dementia and traumatic brain injury (34–37). Meta-analysis of 9 RCT and two phase-IV studies have recently highlighted the efficacy of the multimodal strategy combining Cerebrolysin with standardized rehabilitation therapy for motor and neurological function recovery following AIS (38, 39). Also, the latest safety meta-analysis comprising a total of 12 randomized double-blind trials showed a very good safety profile for patients treated with Cerebrolysin (40). Based on the abovementioned results, Cerebrolysin has been recommended by the European Academy of Neurology and European Federation of Neurorehabilitation Societies and in other practice guidelines as a pharmacological intervention for ischemic stroke, for both the acute- and post-stroke rehabilitation (41–45).

We have hypothesized that adding Cerebrolysin ≤ 8 h following stroke onset in selected patients based on the clinical and radiological criteria (moderate to severe stroke, baseline small ischemic core, good collateral status, significant recanalization following MT) may increase the effectiveness of MT by initiating cytoprotective effects and preventing reperfusion injury and delayed cell death. The multimodal treatment concept of Cerebrolysin combined with MT in AIS and with rehabilitation in post-acute period might also promote and maintain the most effective recovery from stroke. We have chosen Cerebrolysin because of its known pharmacological properties, BBB penetration, good safety profile, parenteral administration, promising preclinical data, and results of RCTs. These factors, together with a multiple-action, multiple-target approach for ischemic stroke could result in a higher likelihood of success in patients with stroke in cerebroprotective studies according to the recommendations of STAIR XI (*Stroke Treatment Academic Industry Roundtable*) conferences (46). Therefore, we aimed to evaluate the efficacy and safety of Cerebrolysin treatment as an add-on therapy to MT in patients with AIS in the early recovery phase in AIS (90 days) and long-term follow-up (12 months).

## Methods and Design

### Study Design and Setting

The study protocol was developed in accordance with the Standard Protocol Items: Recommendations for Interventional Trials (SPIRIT) Statement and the trial was registered on ClinicalTrials.gov on May 27, 2021, with reference number NCT04904341 (47). This academic investigator-initiated study is managed by a group of collaborative clinical researchers and has been planned to investigate health research questions relevant to everyday practice (48, 49). Our main goal was to determine the efficacy and safety of Cerebrolysin treatment as an add-on therapy to MT in reducing global disability in subjects with AIS.

We have planned a single center, prospective, open-label, single-arm study with a 12-month follow-up of consecutive 50 patients referred to the reference stroke center (Military Institute of Medicine, Warsaw, Poland) due to moderate to severe AIS, with a small established infarct core, with good collateral circulation, significant recanalization following MT, and who received additional Cerebrolysin (Cerebrolysin group) compared to 50 historical controls treated with MT alone. The treatment with Cerebrolysin will begin as soon as possible after MT and no later than 8 h following stroke onset. Clinical and radiological evaluation will be performed by blinded assessors. The participants for the historical cohort (control group) will be selected retrospectively from a larger and ongoing, prospectively maintained investigator database from patients who were previously treated in the study center. Database contains imaging, demographic, and outcome data of patients treated with MT by the same team of experienced 3 operators since January 2018. Control patients will be matched one-to-one to patients receiving Cerebrolysin based on the occlusion location (ICA or MCA-M1 or MCA-M2) and then further matched for age (±5 years), baseline mRS (0–2 or 3–5), TICI score (2b or 3), baseline perfusion lesion volume, onset to reperfusion time, and the use of bridging r-tPA using probabilistic matching. If no matched control is identified, we will exclude that case from the primary outcome analysis. Historical controls will receive a similar standard of care with the same diagnostic, endovascular, and perioperative management as Cerebrolysin group and will be excluded if they had an incomplete medical record. All the patients will fulfill the same clinical and radiological inclusion/exclusion criteria.

### Neuroimaging Protocol

According to our neuroradiological acute stroke protocol, all patients qualified for MT receive a noncontrast CT (NCCT) to rule out hemorrhage, CT angiography (CTA), and perfusion CT (CTP) to determine large-vessel occlusion and perfusion status. Follow-up NCCT are acquired at 24 h (all patients) and 30 days (Cerebrolysin group only). NCCT images will be assessed by assessors who are unaware of clinical data. All CT scans are obtained with the patient in a supine position by using a 64-slice GE CT scanner. Early and late ischemic signs will be determined using the Alberta Stroke Program Early CT Score (ASPECTS) scale on baseline and follow-up NCCT (50). CTP is performed after the injection of 50 ml of contrast mean 5 ml/s followed by 50 ml of saline to assess the extension of the core and the ischemic penumbra, which is defined as the mismatch between “mean transit time” and “cerebral blood volume” maps. Collaterals are measured with a multiphase CTA covering the first phase from the carina until the vertex and the second and third phases from the foramen magnum to the vertex. Acquisition is triggered using a bolus tracking (100 HU) in the aortic arch after intravenous injection contrast, and then the second and third phases started 4 s after the previous phase. Collaterals are measured by comparing backfilling arteries beyond the occluded artery to similar arteries in the opposite unaffected hemisphere in three different phases. Intracranial hemorrhages are diagnosed according to the control NCCT scan at 24 h or later in case of neurologic deterioration (an increase of NIHSS score ≥4 points from baseline and parenchymal hematoma type 2 within 36 h of MT). Asymptomatic and symptomatic hemorrhagic transformations and the presence of brain edema with midline shift at 24 h NCCT will also be assessed in study groups because they are a complication of reperfusion therapy, and currently they are regarded as markers of BBB disruption, which may be protected by Cerebrolysin (28, 51, 52). Midline shift defined as any deviation of midline structures (e.g. the septum pellucidum) will be assessed as a dichotomous variable (present or absent).

#### Post-processing Software

Automated processing of NCCT, CTA, and CTP will be performed using the latest CE-marked version of e-Stroke software (Brainomix, Oxford, UK) at baseline, and follow-up imaging will be processed using algorithms in development by Brainomix. This will provide objective and consistent quantification of imaging biomarkers to ensure robust matching to historical controls, as well as the evaluation of imaging endpoints, and will help to reduce the risk of bias when analyzing data. NCCT, CTA and CTP will be processed using e-ASPECTS, e-CTA, and e-CTP modules within e-Stroke respectively (53–58). e-Stroke imaging software is intended to be used as a decision support tool and will be used to facilitate adjudication of patient inclusion criteria for prospective patients and historical controls, where the results are intended for this purpose. MCA vascular enhancement distal to occlusion is rated by using CTA collateral score (CTA-CS, also known as Tan score) (59, 60). On a scale of 0 to 3, higher grades are associated with a better collateral flow (0: absent collateral supply to the occluded MCA territory; 1: collateral supply filling ≤ 50% but >0% of the occluded MCA territory; 2: collateral supply filling >50% but <100% of the occluded MCA territory; 3: 100% collateral supply of the occluded MCA territory). The imaging values that will be derived for each patient using e-Stroke are presented in Table 1.

**Table 1:** Summary of imaging values assessed during the study.

**Baseline**	**Follow-up**
**NCCT**	**NCCT at 24 h**
e-ASPECTS	Final infarct volume (FIV)
e-ASPECTS acute ischemic volume (AIV)	Hemorrhage volume (if any)
Thrombus length on NCCT (if identified)	Midline shift
**CTA**	
CTA collateral score (CTA-CS)	**NCCT at 30 day**
CTA collateral vessel density	Final infarct volume
Location of large vessel occlusion (proximal vs. distal)	
**CTP**	
Ischemic core volume (estimate, relative CBF <30%)	
Hypoperfusion volume (estimate, Tmax >6 s)	
Mismatch ratio	
Mismatch volume	
Alternative estimate of ischaemic core volume (defined using rCBF threshold of <38%)	
Hypoperfusion intensity ratio	

*e-ASPECTS, Alberta Stroke Program Early CT Score; NCCT, non contrast CT; CTA, CT angiography; CBF, cerebral blood flow; CTP, perfusion CT*.

Analysis of baseline imaging, including patient selection and analysis of historical controls is delivered using a deployed installation of the Brainomix e-Stroke software. Analysis involving follow-up imaging will be undertaken by the Brainomix team in the imaging lab and will be performed at the end of the study to ensure consistent biomarkers are derived.

### Thrombectomy Protocol

Patients will be qualified for MT according to the American Heart Association/American Stroke Association (AHA/ASA) and European Stroke Organization—European Society of Minimally Invasive Neurological Therapy (ESO-ESMINT) guidelines (61, 62). Thrombectomy will be performed with any FDA-approved thrombectomy device (stent retriever or aspiration thrombectomy or its combination to achieve safe recanalization) at the discretion of the neurointerventionalist (63, 64). The mechanical thrombectomy procedures were done from groin access by operators familiar with assessed stent-retrievers and who had performed at least 50 endovascular stroke treatment procedures. MT was performed under local or general anesthesia at the discretion of the operator. Eligible patients will be qualified for bridging r-tPA (0.9 mg/kg of rtPA) according to current guidelines (65). Angiographic studies from the endovascular procedure will be assessed for recanalization by two independent raters, who will be unaware of any other imaging and clinical data, and a consensus will be reached in cases of disagreement. The modified treatment in cerebral infarction (mTICI score) will be used to measure the reperfusion grade post thrombectomy (0- no reperfusion; 1-antegrade reperfusion past the initial occlusion, but limited distal branch filling with little or slow distal reperfusion; 2a- antegrade reperfusion of less than half of the occluded target artery previously ischemic territory (e.g., in one major division of the middle cerebral artery (MCA) and its territory); 2b- antegrade reperfusion of more than half of the previously occluded target artery ischemic territory (e.g., in two major divisions of the MCA and their territories); 3-complete antegrade reperfusion of the previously occluded target artery ischemic territory, with absence of visualized occlusion in all distal branches). Successful reperfusion is defined by mTICI score ≥2b.

### Participants

Eligibility criteria (for the active arm and historical control group) are listed in Table 2.

**Table 2:** Study eligibility criteria.

**Clinical inclusion criteria**	**Clinical exclusion criteria**	**Neuroimaging inclusion criteria**	**Neuroimaging exclusion criteria**
Age 18–80 years	Other than AIS serious, advanced, or terminal illness or life expectancy ≤ 6 months	CT ASPECTS ≥6 prior to MT	Acute symptomatic arterial occlusions in more than one vascular territory***
Signs and symptoms consistent with the diagnosis of an anterior circulation AIS	Pre-existing medical, neurological or psychiatric disease that would confound the neurological or functional evaluations **	ICA or MCA-M1 or –M2 occlusion (carotid occlusions can be cervical or intracranial; without tandem MCA lesions) by CTA	Evidence of intracranial tumor (except small meningioma), acute intracranial hemorrhage, neoplasm, or arteriovenous malformation
Stroke onset to groin <6 h*	Pregnancy or lactation	Target mismatch profile on CTP (ischemic core volume <70 ml, mismatch ratio >1.8 and mismatch volume > 15 ml)	Significant mass effect with midline shift
mRS ≤ 1 prior to qualifying stroke (functionally independent for all ADLs)	Known allergy to iodine that precludes an endovascular procedure	Moderate-to-good collateral status on multiphase CTA (>50% MCA territory)	Treatment with another investigational drug within the last 30 days that may interfere with this study's medications
moderate to severe stroke: NIHSS score of ≥5 with presence of any cortical signs (gaze, visual fields, language, or neglect)	Acute or chronic renal failure with calculated creatinine clearance <30 ml/min/1.73 m^2^ or unable to undergo a contrast brain perfusion scan with CT	Effective reperfusion mTICI ≥2b following MT	Patients with nondiagnostic NCCT or CTP maps
Initiation of treatment with Cerebrolysin <8 h following stroke onset (Cerebrolysin group)	Inability to tolerate or comply with study procedures		
Patient has signed the Informed Consent form	Any condition that would represent a contraindication for Cerebrolysin administration (e.g., allergy)		

* *Stroke onset is defined as the time the patient was last known to be at their neurologic baseline (wake-up strokes are eligible if they meet the above time limits)*.

** *E.g., Alzheimer's disease, vascular dementia, Parkinson's disease, demyelinating disease, encephalopathy of any cause,a history of significant alcohol or drug abuse*.

*** *E.g., bilateral MCA occlusions, or an MCA and a basilar artery occlusion*.

#### Treatment

##### Cerebrolysin Treatment (Cerebrolysin Group)

The first Cerebrolysin infusion (30 ml mixed with 250 ml of saline) is intended to be initiated as soon as possible after successful recanalization is achieved and within 8 h of AIS stroke onset. Cerebrolysin treatment will be continued (30 ml/d) once daily until day 21 (first cycle). The patients will receive a second cycle of treatment (30 ml/d for 21 days given in the Outpatient Department or Neurorehabilitation Clinic) from day 69–90 (± 3 days).

##### All Patients (Cerebrolysin Group and Historical Controls)

All patients will receive care in the neurointensive care unit and stroke unit and they will receive a standardized stroke treatment and diagnosis according to the national and international guidelines. The patients will receive iv rt-PA in a 4.5-h window if they meet the accepted criteria. All patients will be assessed for their rehabilitation needs and they will receive rehabilitation (physiotherapy, occupational, and speech therapy) at the Stroke Unit following day 1 until discharge and in Neurorehabilitation Clinic with a minimum of 45 min of physiotherapy 5 days a week and according to local standards (the average rehabilitation duration is 3 months post stroke, with the maximum 4 months).

#### Neurological and Neuropsychological Assessment

Neurological assessments will be based on a routine evaluation scheme performed in both Cerebrolysin and control patients by a senior neurologist before and following MT, at 24 h post MT, at hospital discharge (by mRS and NIHSS), and at 30, 90 days, and 12 months (by mRS, BI, and EQ-5D-5L). The 12-month assessments will be performed through telephone questionnaires which have been shown to have good validity and reliability to on-site assessment. Assessments of language function (by the 15-item Boston Naming Test), hemispatial neglect (by line bisection test), global cognitive function (by The Montreal Cognitive Assessment), and depression (by Hamilton Depression Rating Scale) will be performed at 30 and 90 days by experienced neuropsychologist in only Cerebrolysin group as these tests are not routinely performed in control patients. The assessors will be blinded for the results of the MT. Age, sex, side of lesion, stroke risk factors, time from onset of symptoms to hospital, to CT, to needle, to groin (femoral artery puncture), and to end of the procedure, and adverse events will be collected throughout the study and analyzed with regards to outcome measures.

### Outcome Measures

**The primary outcome measure** will be the overall proportion of subjects receiving Cerebrolysin compared to the control group experiencing a favorable functional outcome (mRS 0–2) at 90 days following stroke onset.

**The secondary objectives** are to determine the efficacy of Cerebrolysin as compared to the control group in reducing the risk of symptomatic and asymptomatic secondary hemorrhagic transformation, brain edema with midline shift, improving neurological outcome (NIHSS 0-2 and mRS at day 7, day 30, and 90); reducing mortality rates (over the 90-day and 12 months study period); and improving: activities of daily living (by BI), health-related quality of life (as measured by the EQ-5D-5L) assessed at day 30, 90, and 12 months. Imaging endpoints will include (at 24 h): final infarct volume; infarct growth (FIV – AIV); infarct growth-CTP (FIV – rCBF <30% volume); and penumbral salvage (1- [Infarct growth-CTP / mismatch volume]). The other measures of efficacy in the Cerebrolysin group will include: changes over time between day 30 and day 90 assessments in language function (by the 15-item Boston Naming Test), hemispatial neglect (by line bisection test), global cognitive function (by The Montreal Cognitive Assessment) and depression (by Hamilton Depression Rating Scale).

### Statistical Analysis

A matched case-control design will be implemented to address the objective of this study. Matching will be blinded to the outcome and will be performed with the use of SPSS 22 algorithm based on prespecified baseline measures, which were selected according to their clinical relevance to stroke outcome. In the case of multiple matches, controls will be selected randomly.

The expected proportions of functional independence are 40 and 75% in the historical controls and Cerebrolysin groups, respectively. Such assumptions are based on the results of MT trials that used CTP for patient selection (ESCAPE, SWIFT PRIME, and substudy of MR CLEAN) (66–68).

Based on the estimated effect size of 35%, a total of 100 patients will be required to test the null hypothesis with an α value of 0.05 and a power of 0.8. The Shapiro–Wilk test will be used to assess the normality of the variables. Continuous variables will be reported as mean±SD if normally distributed or median (interquartile range) if nonparametric. Categorical variables will be reported as proportions. Between groups, comparisons for continuous/ordinal variables will be made with Student *t*-test, Mann–Whitney U-test, ANOVA, paired *t*-test, or Wilcoxon rank sum test, as appropriate. Categorical variables will be compared by χ2 test, Fisher exact test, or McNemar test for discordant pairs, as appropriate. The overall distribution of mRS will be compared between groups (shift in disability levels) using the van Elteren test or Wilcoxon signed-rank test to account for the matching. Binary logistic regression analysis will be performed to identify the predictive factors for functional outcomes. Changes over time in neuropsychological variables in the Cerebrolysin group will be assessed by paired *t*-test. Variables with *P* < 0.1 from the univariate analysis will be included for multivariate logistic regression models. A two-sided *P* < 0.05 will be considered to be statistically significant.

### Study Flow and Timelines

The study flow is summarized in Table 3. All patient identification data will be scrambled to ensure confidentiality. Ethics approval has been received from the Institution Review Board of the Wojskowy Instytut Medyczny w Warszawie, and all patients will have to give informed written consent for the participation in the study. This study will be conducted in accordance with the Declaration of Helsinki. The targeted end date for recruitment is December 2022.

**Table 3:** Study flow and eligibility criteria.

	**Baseline**	**1–21 day**	**7 day**	**30 day**	**69–89 day**	**90 day**	**12 months**
**Cerebrolysin group**							
Eligibility criteria	x						
Informed consent	x						
Cerebrolysin infusion		x			x		
mRS	x		x	x		x	x
NIHSS	x		x	x		x	
BI				x		x	x
EQ-5D-5L				x		x	x
Neuropsychological assessment*				x		x	
Control CT, CTP		24 h post MT**		x			
Neurorehabilitation		x		x	x	x	
**Historical control group**	
mRS	x		x	x		x	x
NIHSS	x		x	x		x	
BI				x		x	x
EQ-5D-5L				x		x	x
Control CT		24 h post MT**					
Neurorehabilitation		x		x	x	x	

* *The 15-item Boston Naming Test, line bisection test, the Montreal Cognitive Assessment, Hamilton Depression Rating Scale*.

** *NCCT*.

## Discussion

The study will investigate whether combining cytoprotection with reperfusion therapy may modulate stroke recovery with a view to describing the optimal treatment window (acute and postacute phase of stroke). We will evaluate clinical and imaging markers for potential use in future clinical trials on cytoprotection. The results of this pilot study will not only shed light on the potential efficacy of Cerebrolysin as an adjunct treatment for AIS but will be essential in shaping a further double-blind RCT on Cerebrolysin or will supplement ongoing larger-scale projects on other neuroprotection agents in both acute and postacute stroke patients. Although Cerebrolysin is used clinically in over 50 European and Asian countries, it has not been approved for use in the USA or Australia. Therefore, more robust clinical trials with a greater number of participants are needed to clarify the clinical application of Cerebrolysin as a monotherapy and in combination with other therapeutics (69).

### Limitations of the Study

Lack of randomization is the main limitation of the present research. We acknowledge that the studied group is small, and a historical control group potentially may introduce multiple biases compared to a concurrent control. However, as it is a proof-of-concept study, we believe these biases may be minimized and become acceptable by careful choice of controls fulfilling the same selection criteria, having similar prognostic factors, and subjected to the same procedures from stroke onset to 12 months of follow-up (70). Adjustments for well-known prognostic factors and simple endpoints used in this study will also help to reduce the complexity and subjectivity of the assessment (71). We decided to use the historical controls because we have a recent, large (containing more than 300 records), broad-based local dataset which we are obliged to conduct and provide to the National Health Found, which contains high quantity and quality clinical, neuropsychological, and radiological data of patients treated with MT and followed-up for 12 months (72). There are also some advantages of using carefully chosen historical data, such as costs and enrolment time can be cut dramatically and more resources can be allocated to the experimental group (73). We also believe the results of the current study will supplement the current knowledge about neuroprotection use in AIS and will enable us to conduct a further trial with the use of a PROBE design to avoid part of these biases.

The therapeutic time delay over 6 h may weaken the efficacy of Cerebrolysin on the neurologic functional recovery; however based on the current practice in our center, we expect that at least 50% of subjects are to receive Cerebrolysin in <6 h post-stroke onset. Penumbra imaging could select patients with better rates of spontaneous recovery because of collateral circulation, thereby reducing the power to detect a benefit of neuroprotection, but on the other hand, the lack of reperfusion reduces substantially the power of a stand-alone neuroprotection trial to detect treatment effects. Also, the addition of advanced imaging to identify a “responder” population leads to a reduced sample size (74). Another limitation is that the study population is limited to patients recruited from one center, which restrains the generalizability of the results to other populations. There is also a lack of robust imaging endpoints to study the presumed biological effects of Cerebrolysin; however we will evaluate hemorrhagic transformation and cerebral edema by midline shift, both of which are associated with BBB permeability. For these limitations, further studies are therefore warranted.

### Strengths and Relevance

The presented study has several advantages. First, there is a lack of clinical trials on cytoprotective agents in combination with reperfusion therapy in AIS. Preliminary results from different studies indicate a high potential for some neuroprotective treatments in addition to reperfusion; however more data is needed (75). Second, Cerebrolysin has been regarded as an ideal agent for functional recovery after AIS because of its multiple attributes including cytoprotective properties and neurotrophic activity. Although MT and Cerebrolysin treatment can be beneficial in patients with large ischemic core, for the purpose of the current study and to minimize subject heterogeneity, we have decided to include patients based on clinical and neuroradiological criteria and with criteria that are validated by other trials on MT. Importantly, we have planned a long-term follow-up of 12 months, which will enable us to evaluate both short- and long-term outcomes. The study methodology minimizes heterogeneity through imaging-based selection and ensures that the neuroprotective effect is amplified through reperfusion. We believe that previously tested cytoprotective drugs warrant re-evaluation since they were tested in studies where LVO recanalization was rarely achieved. Further investigation of the clinical effects of Cerebrolysin as an add-on therapy to reperfusion therapy is therefore reasonable because there is an unmet need for neuroprotective or neurotrophic drugs with good efficacy in neurological functional recovery in AIS patients.

## Ethics Statement

The studies involving human participants were reviewed and approved by Institution Review Board of the Wojskowy Instytut Medyczny w Warszawie (No. 53/WIM/2020). The patients/participants provided their written informed consent to participate in this study.

## Author Contributions

JS, PP, AS, and AM are on the Scientific Committee for the current project. All authors were involved in the design of the protocol, revised the draft, and approved the final manuscript.

## Funding

The study was granted by the internal scientific grant by the Wojskowy Instytut Medyczny w Warszawie (No. 00589).

## Conflict of Interest

ZC, DB, BK, RN, and GH were employed by Brainomix Ltd. The remaining authors declare that the research was conducted in the absence of any commercial or financial relationships that could be construed as a potential conflict of interest.

## Publisher's Note

All claims expressed in this article are solely those of the authors and do not necessarily represent those of their affiliated organizations, or those of the publisher, the editors and the reviewers. Any product that may be evaluated in this article, or claim that may be made by its manufacturer, is not guaranteed or endorsed by the publisher.
